# Pancaost syndrome related to hydatid cyst

**DOI:** 10.11604/pamj.2013.14.118.1754

**Published:** 2013-03-27

**Authors:** Ibrahim Dao, Brahim El Mostarchid, Justin Onen, Cherkaoui Mandour, Cherif Abad El Asri, Mohammed Boucetta

**Affiliations:** 1 Department of Neurosurgery, Mohammed V Military Teaching Hospital, University of Mohammed V-Souisi; 2 Department of Neurosurgery, Hopital des specialités Rabat, University of Mohammed V-Souisi

**Keywords:** Pancoast syndrome, hydatid cyst, Horner's syndrome, lung apex

## Abstract

Pancoast syndrome remains a rare presentation of pulmonary diseases. Even in endemic area of echinococcosis, lung's hydatid cyst is seldomly revealed by this syndrome. We report a case of a 38 year old male patient who presented to our department with neck and left superior limb pain associated with palmar muscle atrophy and Horner's syndrome. Radiological investigations suggested the diagnosis of hydatid cyst of the left lung apex which was confirmed by surgical excision and pathological examination of the lesion. This case highlights an uncommon etiology of Pancoast syndrome which might mislead physicians in their practice.

## Introduction

Pancoast was the first author to describe in 1924 a syndrome related to tumors that involve the lung apex [1]. This syndrome is commonly reported in neoplasm diseases and accounts for only 3% to 5% of all lung cancer presentations [1]. Although pulmonary hydatid cyst is still widespread throughout the world and remains an important public health issue in some regions (such as Middle Eastern, Turkey, South America and around Mediterranean borders) [2], it is very scarcely involved in the etiologies of this so called Pancoast syndrome. We report this uncommon case of Pancoast syndrome related to lung apex hydatid cyst occurring in a 38 year- old man who successfully underwent surgery in our department.

## Patient and observation

**Figure 1:**
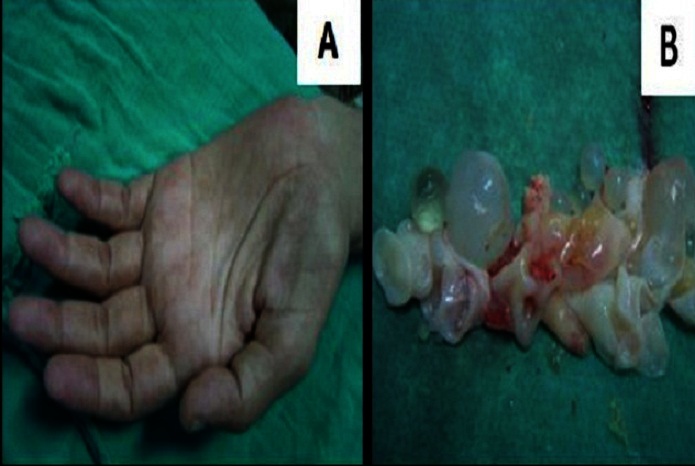
A: Our patient's left hand showing an atrophy of thenar and hypothenar muscles; B: some hydatid cysts which have been removed

This 38 year old immunocompetent man coming from a rural area was followed in our department for vertebral hydatid cyst disease for 4 years. He had been operated twice for thoracic and lumbar hydatid cyst revealed by recurrent paraplegia. He complained of a two month history of cervical and shoulder pain radiating downward toward the ulnar side of his left arm and forearm reaching the fourth and fifth fingers. Physical examination revealed decreased muscular strength in the fingers mainly in the last fingers of the left hand associated with thenar and hypothenar muscle atrophy (Figure 1). Horner's syndrome of the left eye, namely a miosis, ptosis and enophtalmos with ipsilateral facial anhidrosis was also noticeable.

**Figure 2:**
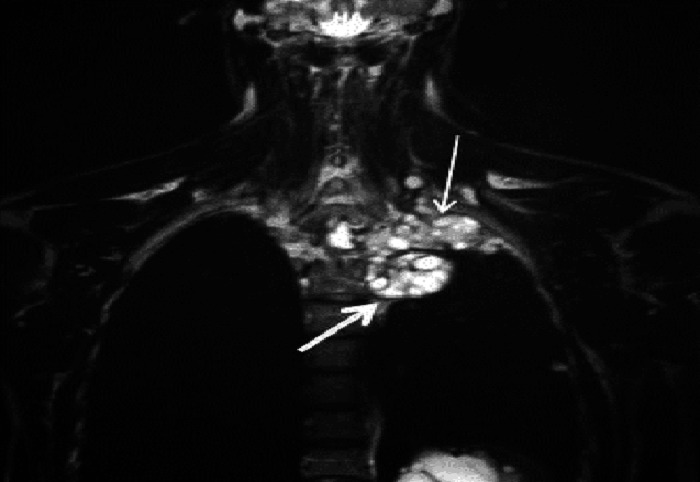
Coronal T2 weighted MRI showing hydatid cysts of the pulmonary apex (thin arrow) delineated at the inferior border by an hypointense rim (thick arrow)

Radiological examination consisting of magnetic resonance imaging (MRI) showed a multi vesicular lesion of the left pulmonary apex which was hypointense on T1 weighted images. This lesion was hyperintense on T2 weighted images and delineated by a hypointense rim with a “bunch of grapefruit” appearance (Figure 2). There was also an invasion of the first and second thoracic vertebrae as well as the corresponding ribs. Serologic test results for hydatid cyst were positive. The patient was operated with the assistance of thoracic surgeons through a left Paulson thoracotomy. Post operative period was uneventful and adjuvant Albendazole based chemotherapy was administrated.

## Discussion

Pancoast syndrome has been defined as a constellation of characteristic symptoms and signs that includes shoulder and arm pain along the distribution of the eighth cervical nerve trunk and the first and second thoracic nerve trunks, Horner syndrome, and weakness and atrophy of the muscles of the hand, most commonly caused by local extension of an apical lung tumor at the superior thoracic inlet [3, 1]. However, this clinical condition is very rarely associated with a pulmonary apex hydatid cyst including endemic area of echinoccosis [2, 4]. Like in our case, there is a good correlation between the clinico radiological presentation and the anatomic involvement [5]. Indeed, this thoracic inlet space is limited by the first thoracic vertebra backward, the sternum forward, the first two ribs on each side and contains the brachial plexus namely its lower trunk, the stellate ganglion and the parietal pleura capping the lung's apex. This region is also crossed by the phrenic nerve, the vagus nerve with its recurrent laryngeal branch and subclavian vessels [5, 1, 6].

The shoulder pain in our patient was clearly explained by the hydatid cyst invasion of not only the brachial plexus, but also the endothoraic fascia and parietal pleura (Figure 2). This pain radiated downward to the medial side of the forearm and to the fourth and fifth fingers which corresponds to the distribution of ulnar nerve that originates from the lower trunk of the brachial plexus formed by C8 and T1 nerve roots. Moreover, the weakness and atrophy of the hypothenar muscles of the left hand was related to this invasion of ulnar nerve that innervates these muscles (Figure 1).

Clinical symptoms of phrenic or recurrent laryngeal nerve involvement and superior vena cava syndrome are uncommon in Pancoast syndrome and were absent in our case [5, 1].

Pulmonary involvement symptoms such as cough, dyspnea and hemoptysis are seldom too, mainly in the initial stage of the disease but may occur later in the majority of patients [5, 1]. These symptoms were not seen in our patient.

Horner's syndrome consisting of ipsilateral miosis, ptosis, enophtalmous and facial anhidrosis was present in our case. It is caused by a direct pressure of the hydatid cysts on the sympathetic chain above all the stellate ganglion medial to the apex. Classic chest radiography and computed tomography (CT) are the main tools for radiologic diagnosis [2].

Chest radiography usually shows a well defined homogenous opacity on the apex although this feature is indistinguishable from other mass lesions [8]. However, CT scan diagnosis has an accuracy of 98% [4] and shows a non enhanced water density lesion with well delineated borders and may demonstrate the daughter cysts [4, 7]. Magnetic resonance imaging (MRI) is more helpful for the investigation of the relation of hydatid cyst with soft tissues [4]. On MRI, hydatid cysts are hypointense on T1 weighted images and hyperintense on T2 weighted images with sometimes a rim sign which consists of a low signal intensity rim surrounding the cyst and corresponding to the pericyst [7, 8]. This hypointense rim was clearly seen in our case (Figure 2). Since it is a parasite disease, eosinophilia is present almost in 25% and serological diagnosis's sensitivity ranges between 40 and 90%. Serological diagnosis is more helpful in the diagnosis of reccurences [2, 4] and was positive in our case. The differential diagnosis above all in uncommon radiological aspect or complicated hydatid cyst includes other etiologies of Pancoast's syndrome mainly tumors, but also hematologic conditions, infectious diseases, neurogenic thoracic outlet syndromes (such as the cervical rib syndrome), and pulmonary amyloid nodule [1, 5].

Because they are “benign” lesions, hydatid cysts resulting in Pancoast syndrome require surgical management to decompress neurologic structures by total removal of the cysts as soon as possible [3, 2, 4, 7] and Paulson thoracotomy seems to be suitable for some authors [2]. Our patient has been operated through this approach.

Adjuvant chemotherapy with Albendazole is required for patients at high risque of recurrence (those with multiple hydatid cysts in the thoracic cavity, multiple organ cysts, or ruptured hydatid cyst). Therefore, our patient received this chemotherapy [3, 2].

## Conclusion

Pulmonary apex hydatid cyst should be included in the differential diagnosis of Pancoast syndrome especially in patients coming from endemic regions of Echinococcosis.
